# Remdesivir Effectiveness in Reducing the Risk of 30-Day Readmission in Vulnerable Patients Hospitalized for COVID-19: A Retrospective US Cohort Study Using Propensity Scores

**DOI:** 10.1093/cid/ciae511

**Published:** 2024-10-15

**Authors:** Essy Mozaffari, Aastha Chandak, Robert L Gottlieb, Andre C Kalil, Heng Jiang, Thomas Oppelt, Mark Berry, Chidinma Chima-Melton, Alpesh N Amin

**Affiliations:** Medical Affairs, Gilead Sciences, Foster City, California, USA; Evidence and Access, Certara, New York, New York, USA; Department of Internal Medicine, Baylor University Medical Center, Dallas, Texas, USA; Baylor Scott & White Heart and Vascular Hospital, Dallas, Texas, USA; Baylor Scott & White The Heart Hospital, Plano, Texas, USA; Baylor Scott & White Research Institute, Dallas, Texas, USA; Division of Infectious Diseases, Department of Internal Medicine, University of Nebraska Medical Center, Omaha, Nebraska, USA; Evidence and Access, Certara, Paris, France; Medical Affairs, Gilead Sciences, Foster City, California, USA; Real World Evidence, Gilead Sciences, Foster City, California, USA; Pulmonary Division, Tele-ICU Inc, Los Angeles, California, USA; Division of Hospital Medicine & Palliative Medicine, Department of Medicine, University of California Irvine, Orange, California, USA

**Keywords:** COVID-19, SARS-CoV-2, readmission, inverse probability of treatment weighting, remdesivir, elderly, immunocompromised, data science, propensity scores, comorbidity, real-world evidence, omicron

## Abstract

**Background:**

Reducing hospital readmission offer potential benefits for patients, providers, payers, and policymakers to improve quality of healthcare, reduce cost, and improve patient experience. We investigated effectiveness of remdesivir in reducing 30-day coronavirus disease 2019 (COVID-19)-related readmission during the Omicron era, including older adults and those with underlying immunocompromising conditions.

**Methods:**

This retrospective study utilized the US PINC AI Healthcare Database to identify adult patients discharged alive from an index COVID-19 hospitalization between December 2021 and February 2024. Odds of 30-day COVID-19-related readmission to the same hospital were compared between patients who received remdesivir vs those who did not, after balancing characteristics of the two groups using inverse probability of treatment weighting (IPTW). Analyses were stratified by maximum supplemental oxygen requirement during index hospitalization.

**Results:**

Of 326 033 patients hospitalized for COVID-19 during study period, 210 586 patients met the eligibility criteria. Of these, 109 551 (52%) patients were treated with remdesivir. After IPTW, lower odds of 30-day COVID-19-related readmission were observed in patients who received remdesivir vs those who did not, in the overall population (3.3% vs 4.2%, respectively; odds ratio [95% confidence interval {CI}]: 0.78 [.75–.80]), elderly population (3.7% vs 4.7%, respectively; 0.78 [.75–.81]), and those with underlying immunocompromising conditions (5.3% vs 6.2%, respectively; 0.86 [.80–.92]). These results were consistent irrespective of supplemental oxygen requirements.

**Conclusions:**

Treating patients hospitalized for COVID-19 with remdesivir was associated with a significantly lower likelihood of 30-day COVID-19-related readmission across all patients discharged alive from the initial COVID-19 hospitalization, including older adults and those with underlying immunocompromising conditions.

## Teaser

Clinical benefits of remdesivir continues to extend into the Omicron era by conferring significantly lower odds of 30-day coronavirus disease 2019 (COVID-19)-related hospital readmission across all patients hospitalized for COVID-19, including vulnerable older adults and those with underlying immunocompromising conditions.

The coronavirus disease (COVID-19) pandemic is no longer a “public health emergency,” yet it remains a clinically consequential infectious disease in the United States with more attributable deaths than seasonal influenza and requires timely medical attention [1, 2]. Given the broad spectrum of clinical manifestations, the recovery course of COVID-19 differs based on the severity of the infection, ranging from a few days for mild symptomatic infection to weeks or months for severe or critical infection [3] and may require hospitalization in some cases. In general, higher rates of hospitalizations are reported in older patients (ie, >50 years of age and especially those ≥65 years of age), immunocompromised patients, and patients with comorbidities [4, 5].

Recent literature indicates that a considerable proportion of patients with COVID-19 are subsequently readmitted to the hospital after the patient's initial COVID-19-related hospitalization (index hospitalization) [6, 7]. Notably, the 30-day hospital readmission rate due to COVID-19 has been reported to be between 7.1% and 14.5% in 2020–2021 [8–10], thereby adding to the clinical, economic, and healthcare utilization burden as well as impacting the patient's ability to return to their health baseline.

Unplanned hospital readmission across the breadth of medical conditions is a core quality metric of healthcare provision [11]. It imposes a considerable financial and health-related burden on patients, caregivers, and the healthcare system [12]. Across all payers, the overall readmission rate following any cause of hospitalization is estimated to be 14.0% [13], costing billions of dollars annually in addition to the cost associated with index admissions [14, 15]. Reducing hospital readmission has been a national priority for providers, payers, and policymakers to improve quality of healthcare, reduce healthcare costs, and improve patient experience [15–18].

Remdesivir, the first antiviral drug approved for treatment of COVID-19 [19–25], is recommended for initiation among patients with mild-to-moderate COVID-19 (ambulatory or hospitalized) at high risk of progressing to severe COVID-19. Remdesivir is also recommended for use in patients hospitalized with hypoxemic COVID-19, including patients requiring low flow oxygen (LFO) as an adjunct to corticosteroids, and as an adjunct to corticosteroids and an additional immunomodulatory agent for many patients requiring high flow oxygen (HFO)/non-invasive ventilation (NIV) [4, 26–30]. Multiple studies have suggested that treatment with remdesivir was associated with reduced risk of hospital readmission; at 30, 60, and 90 days irrespective of the severe acute respiratory syndrome coronavirus 2 variant [30], in immunocompromised individuals [31, 32], and across a broad spectrum of COVID-19 hospitalized patients [23, 33, 34]. Despite remdesivir's standing as the antiviral standard of care for use in patients hospitalized for COVID-19, and its observed association with improved survival [19, 34–36] and readmission outcomes [23, 24, 31–33, 37], current updated evidence, including real-world evidence are instrumental to further inform clinical decision making in the evolving endemic era, specifically with the evolving Omicron subvariants, which were not examined in prior research. Additionally, there remains an ongoing need to better understand optimal treatment and improve outcomes among vulnerable patient populations such as the elderly and less frequently researched subgroups such as those with immunocompromising conditions.

Using a geographically diverse hospital database in the United States, we extend previous evidence of the effectiveness of remdesivir at reducing hospital readmission to the current endemic. In this study, we examined the association between remdesivir treatment during the initial hospitalization for COVID-19 and the likelihood of 30-day COVID-19-related readmission during the Omicron-predominant era, including the sub-populations of older adults and those with immunocompromising conditions who were discharged alive from the initial COVID-19 hospitalization.

## METHODS

### Study Design and Data Source

This retrospective comparative effectiveness study extracted hospitalization records from the US PINC AI Healthcare Database (PHD, formerly Premier Healthcare Database; www.pinc-ai.com), a large, geographically diverse, Health Insurance Portability and Accountability Act compliant, all-payer hospital administrative billing database that contains approximately 25% of all inpatient hospitalizations in the United States. This database captures information on patient demographics, procedures and medications administered, disease states, costs and resource utilization, and clinical outcomes. Patients can be followed in the same hospital across inpatient and hospital-based outpatient settings, and their length of stay and readmissions to the same hospital can be measured [38].

### Study Population

Patients ≥18 years of age, hospitalized for COVID-19, with a primary discharge diagnosis code of COVID-19 (International Classification of Diseases, 10th revision, Clinical Modification [ICD-10-CM] code U07.1), flagged as “present-on-admission” and discharged alive between December 2021 and February 2024 were identified in the database. Patients who were discharged within 30 days of the end of the study period (February 2024) were also excluded so that all patients discharged alive would have at least 30 days of follow-up post-discharge. Patients were excluded if they met any of the following criteria: discharge documented as “expired” or “transfer to hospice,” pregnant, had incomplete records, transfer to or from another hospital, admission for elective procedures, or use of extracorporeal membrane oxygenation (ECMO) during the index COVID-19 hospitalization. Furthermore, as stratification by the requirement for supplemental oxygenation in the index admission is a key requirement of our analysis, only hospitals affirmatively identified to place charges for supplemental oxygen independently of room charges were included (a method validated previously via sensitivity analyses [33]).

Eligible patients were divided into 2 groups: those treated with remdesivir and those not treated with remdesivir during the index COVID-19 hospitalization. Patients treated with remdesivir received at least a single dose of remdesivir during their index hospitalization. In contrast, patients not treated with remdesivir did not receive remdesivir during the index hospitalization. Sub-populations analyzed included all eligible adults hospitalized for COVID-19 (overall), elderly population (≥65 years of age), and patients with an underlying immunocompromising condition (identified using the ICD-10-CM codes for specified immunocompromising conditions; codes listed in Supplementary Table 1). Each of these subgroups were further characterized based on supplemental oxygen requirements (no supplemental oxygen charges [NSOc] vs any supplemental oxygen charges [SOc]).

### Study Outcomes and Covariates

The primary outcome was COVID-19-related hospital readmission defined as readmission to the same hospital with a primary or secondary discharge diagnosis of COVID-19 within 30 days of index hospitalization. A readmission to a different hospital in the database or to a hospital not part of the database is not captured in this database.

Our study also captured the following covariates for index COVID-19 hospitalization, including demographics (age group, gender, race, ethnicity), admission month, Charlson Comorbidity Index (CCI), comorbid conditions of interest (cancer, immunocompromising condition, obesity, chronic obstructive pulmonary disorder (COPD), cardiovascular disease, diabetes mellitus, and renal disease) hospital characteristics (hospital bed size, teaching status, region, urban/rural setting), supplemental oxygen requirements (NSOc and any SOc), and intensive care unit (ICU) stay, and use of corticosteroids (Supplementary Table 1). Covariates such as these are considered clinically relevant for COVID-19-related outcomes as they represent differences in patient demographics, disease severity, and hospital-related variations of care.

For the readmission hospitalization, the following covariates were captured: primary diagnosis of COVID-19, COVID-19 flagged as “present-on-admission,” primary or secondary diagnosis of pneumonia due to COVID-19 (ICD-10-CM code J12.82), length of stay at readmission, use of inpatient COVID-19 treatments (remdesivir monotherapy, corticosteroids monotherapy, remdesivir as an adjunct to corticosteroids alone or an additional immunomodulatory agent like baricitinib/tocilizumab), and all-cause in-hospital mortality (discharge status of “expired” or “hospice”).

### Statistical Analysis

Patient characteristics were analyzed descriptively for the comparison groups treated with and not treated with remdesivir during their index COVID-19 hospitalization for the overall population, older adults, and those with an underlying immunocompromising condition (Supplementary Table 1).

Given the underlying differences in the 2 treatment groups, the inverse probability of treatment weighting (IPTW) approach was used to adjust for potential confounding and allow for a scientifically robust comparative assessment of the differences in the outcomes between the treatment groups. Propensity scores (PSs) represent the probability of receiving the treatment of interest. In this study, PSs were estimated using separate logistic regression models with exposure to remdesivir as the dependent variable for the 2 supplemental oxygen requirement categories (NSOc vs SOc) and included baseline covariates (age group, gender, race, ethnicity, corticosteroid use, CCI, specific comorbid conditions of cancer, immunocompromising condition, obesity, COPD, cardiovascular disease, diabetes mellitus, and renal disease, admission month, supplemental oxygen requirement, ICU use, hospital bed size, and hospital setting, hospital teaching status, and hospital region). The separate models were used to ensure valid comparability within the supplemental oxygen groups. Furthermore, separate PSs were computed for each of the 3 patient cohorts analyzed in this study. Patients treated with remdesivir and those who did not receive remdesivir during index hospitalization were balanced using IPTW derived from the computed PSs [39]. Extreme PSs, ie, < 0.05 and >0.95 were trimmed. The likelihood of 30–day COVID-19-related readmission to the same hospital, after IPTW for remdesivir and nonremdesivir groups was estimated using multivariable logistic regression analysis. For 3 study cohorts, analyses were performed overall, and by supplemental oxygen requirement during the index admission (NSOc and any SOc). Odds ratios (OR) and 95% confidence intervals (CIs) were summarized. All statistical analyses were conducted using SAS Version 9.4 (SAS 9.4, SAS Institute Inc., Cary, North Carolina, USA). All statistical tests were 2-sided, and the level of significance was *P* ≤ .05 without adjustment for multiple testing. Furthermore, characteristics of the readmission hospitalization were described post-IPTW.

## RESULTS

**Figure 1.**
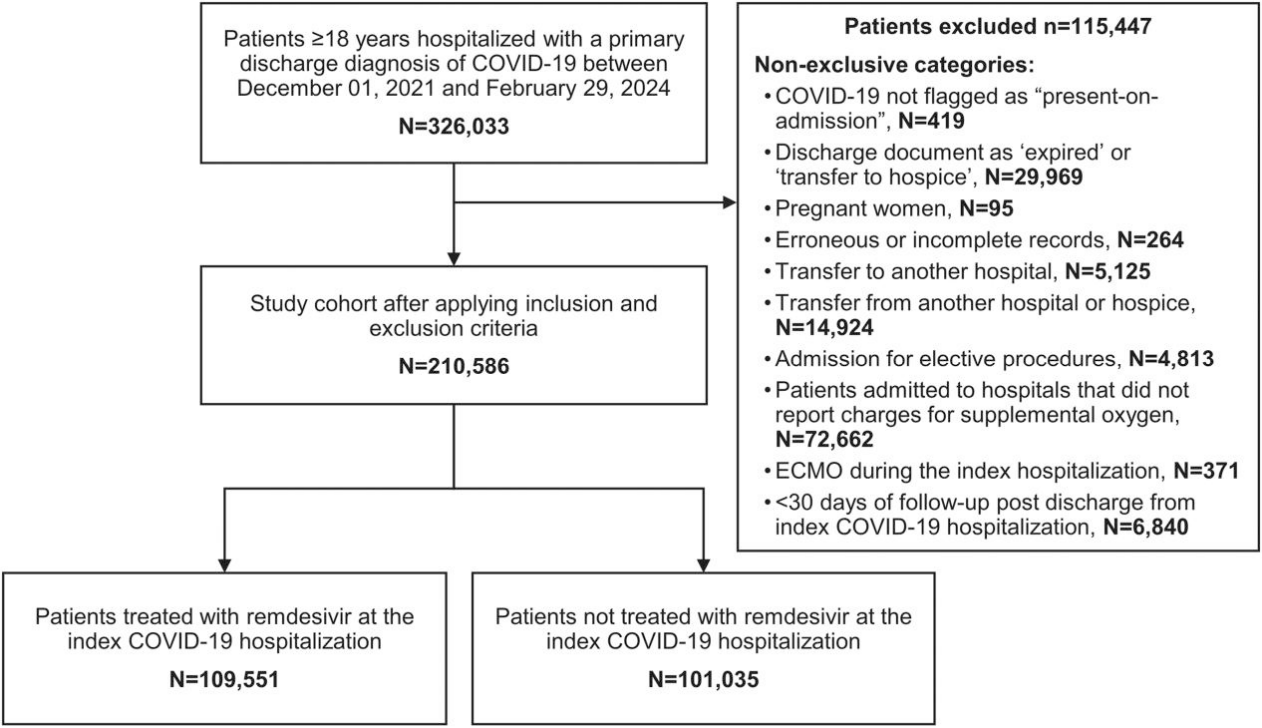
Study flow diagram. Abbreviations: COVID-19, coronavirus disease 2019; ECMO, extracorporeal membrane oxygenation.

A total of 326 033 patients were hospitalized for COVID-19 between December 2021 and February 2024; of these, 210 586 patients met the eligibility criteria (Figure 1).

Patient characteristics at index hospitalization were balanced post-IPTW with an absolute standardized mean difference <0.15 between the 2 groups in the overall, elderly, and immunocompromised populations (Table 1, Supplementary Tables 2 and 3, and Supplementary Figures 1–3).

**Table 1.** Characteristics of Patients During the Index COVID-19 Hospitalization in Overall Population (Before and After IPTW)

	Before IPTW			After IPTW^a^		
	NonRemdesivir	Remdesivir	SMD	NonRemdesivir	Remdesivir	SMD
Number of patients	n = 101 035	n = 109 551				
Number of hospitals	n = 927	n = 925				
**Age, y (not included in PS calculation)**						
Mean (SD)	69.7 (15.6)	70.0 (14.9)	0.01	69.7 [21.9]	70.2 [20.9]	0.02
Median (Q1, Q3)	73.0 (61.0, 82.0)	72.0 (61.0, 82.0)		72 [61, 81]	73 [61, 82]	
**Age group, y**						
18–49	11 349 (11.2)	11 124 (10.2)	0.04	10.5	10.3	0.00
50–64	20 486 (20.3)	23 535 (21.5)		20.9	20.8	
≥65	69 200 (68.5)	74 892 (68.4)		68.6	68.9	
**Race**						
White	75 336 (74.6)	83 648 (76.4)	0.11	75.5	75.5	0.00
Black	16 122 (16.0)	14 681 (13.4)		14.6	14.7	
Asian	1930 (1.9)	2647 (2.4)		2.2	2.2	
Other	7647 (7.6)	8575 (7.8)		7.7	7.6	
**Gender**						
Female	52 364 (51.8)	55 824 (51.0)	0.04	51.2	51.2	0.00
**Ethnicity**						
Hispanic	8374 (8.3)	11 650 (10.6)	0.15	9.4	9.4	0.00
Non-Hispanic	85 146 (84.3)	91 869 (83.9)		84.3	84.4	
Unknown	7515 (7.4)	6032 (5.5)		6.3	6.2	
**CCI**						
0	19 018 (18.8)	18 954 (17.3)	0.08	17.8	17.5	0.00
1–3	52 948 (52.4)	61 280 (55.9)		54.2	54.2	
≥4	29 069 (28.8)	29 317 (26.8)		28.0	28.3	
**Comorbid conditions**						
Immunocompromising condition	14 775 (14.6)	18 542 (16.9)	0.06	16.0	16.2	0.00
Cancer	5906 (5.8)	7684 (7.0)	0.05	6.5	6.6	0.00
Obesity	25 522 (25.3)	31 521 (28.8)	0.08	27.1	27.1	0.00
COPD	32 448 (32.1)	41 668 (38.0)	0.12	35.4	35.5	0.00
Cardiovascular disease	86 520 (85.6)	93 354 (85.2)	−0.01	85.5	85.7	0.00
Diabetes mellitus	38 276 (37.9)	41 606 (38.0)	0.00	38.1	38.1	0.00
Renal disease	29 404 (29.1)	26 377 (24.1)	−0.11	26.8	27.1	0.01
**Maximum supplemental oxygen requirement**						
IMV/ECMO	2293 (2.3)	2863 (2.6)	0.33	2.5	2.5	0.06
HFO/NIV	11 684 (11.6)	21 015 (19.2)		15.7	15.6	
LFO	28 856 (28.6)	40 649 (37.1)		32.9	33.2	
NSOc	58 202 (57.6)	45 024 (41.1)		48.8	48.7	
**ICU use**	14 779 (14.6)	22 128 (20.2)	0.15	17.7	17.8	0.00
**Use of corticosteroids**	64 374 (63.7)	95 110 (86.8)	−0.01	75.9	76.1	0.00
**Hospital setting**						
Rural	13 378 (13.2)	13 376 (12.2)	0.09	12.7	12.5	−0.01
Urban	87 657 (86.8)	96 175 (87.8)		87.3	87.5	
**Hospital teaching status**	41 028 (40.6)	46 809 (42.7)	0.09	41.8	41.9	−0.01
**Hospital census region**						
Midwest	25 608 (25.3)	26 466 (24.2)	0.14	25.0	25.1	0.00
Northeast	12 128 (12.0)	17 898 (16.3)		14.3	14.2	
South	52 760 (52.2)	51 643 (47.1)		49.1	49.0	
West	10 539 (10.4)	13 544 (12.4)		11.6	11.7	
**Hospital bed size**						
<100	8908 (8.8)	9210 (8.4)	0.09	8.7	8.6	0.00
100–199	17 084 (16.9)	19 447 (17.8)		17.2	17.1	
200–299	21 639 (21.4)	21 799 (19.9)		20.5	20.6	
300–399	18 440 (18.3)	17 507 (16.0)		16.8	16.8	
400–499	11 061 (10.9)	11 702 (10.7)		11.1	11.1	
≥500	23 903 (23.7)	29 886 (27.3)		25.7	25.7	
**Admission month**						
Dec 2021	11 125 (11.0)	15 247 (13.9)	0.15	12.4	12.2	0.00
Jan 2022	20 393 (20.2)	22 075 (20.2)		20.0	19.7	
Feb 2022	5546 (5.5)	4918 (4.5)		5.0	4.9	
Mar 2022	1071 (1.1)	993 (0.9)		1.0	1.0	
Apr 2022	1062 (1.1)	1144 (1.0)		1.0	1.1	
May 2022	2848 (2.8)	3124 (2.9)		2.8	2.8	
Jun 2022	3919 (3.9)	3843 (3.5)		3.6	3.6	
Jul 2022	5611 (5.6)	5234 (4.8)		5.1	5.1	
Aug 2022	4808 (4.8)	4433 (4.0)		4.4	4.4	
Sep 2022	3296 (3.3)	3055 (2.8)		3.0	3.1	
Oct 2022	2709 (2.7)	2734 (2.5)		2.6	2.6	
Nov 2022	3055 (3.0)	3150 (2.9)		3.0	3.0	
Dec 2022	4918 (4.9)	5319 (4.9)		4.9	4.9	
Jan 2023	4094 (4.1)	4173 (3.8)		3.9	4.0	
Feb 2023	2451 (2.4)	2680 (2.4)		2.5	2.5	
Mar 2023	2058 (2.0)	2171 (2.0)		2.0	2.1	
Apr 2023	1407 (1.4)	1485 (1.4)		1.4	1.4	
May 2023	991 (1.0)	1167 (1.1)		1.0	1.0	
Jun 2023	819 (0.8)	836 (0.8)		0.8	0.8	
Jul 2023	1180 (1.2)	1226 (1.1)		1.1	1.2	
Aug 2023	2390 (2.4)	2623 (2.4)		2.4	2.4	
Sep 2023	2709 (2.7)	2944 (2.7)		2.7	2.7	
Oct 2023	2038 (2.0)	2335 (2.1)		2.1	2.1	
Nov 2023	2333 (2.3)	2774 (2.5)		2.5	2.5	
Dec 2023	3642 (3.6)	4481 (4.1)		3.9	4.0	
Jan 2024	3072 (3.0)	3664 (3.3)		3.2	3.3	
Feb 2024	1490 (1.5)	1723 (1.6)		1.5	1.6	
**Length of stay, d (not included in PS calculation)**						
Mean (SD)	6.0 (12.9)	7.1 (11.6)	0.09	6.3 (20.0)	6.8 (15.4)	0.03
Median (Q1, Q3)	4.0 (2.0, 7.0)	5.0 (3.0, 8.0)		4 (2.0, 7.0)	5.0 (3.0, 7.0)	
**Remdesivir treatment duration, d (not included in PS calculation)**						
Mean (SD)	-	5.3 (2.3)	-	-	5.2 (3.2)	-
Median (Q1, Q3)	-	5.0 (4.0, 6.0)	-	-	5.0 (4.0, 6.0)	-
**Hospital day of remdesivir initiation, d (not included in PS calculation)**						
Mean (SD)	-	1.4 (0.8)	-	-	1.4 (1.1)	-
Median (Q1, Q3)	-	1.0 (1.0, 2.0)	-	-	1.0 (1.0, 2.0)	-

Data are presented as n (%) before IPTW and as % after IPTW, unless otherwise indicated.

Abbreviations: CCI, Charlson comorbidity index; COPD, chronic obstructive pulmonary disorder; COVID-19, coronavirus disease 2019; d, day(s); ECMO, extracorporeal membrane oxygenation; HFO, high-flow oxygen; ICU, intensive care unit; IMV, invasive mechanical ventilation; IPTW, inverse probability treatment weighting; LFO, low-flow oxygen; NIV, non-invasive ventilation; NSOc, no supplementary oxygen charges; PS, propensity score; Q, quarter; SD, standard deviation; SMD, standardized mean difference.

^a^After trimming extreme propensity scores <0.05 and >0.95.

### Overall Population

Of the 210 586 patients, 109 551 (52.02%) were treated with remdesivir, and 101 035 (47.98%) patients did not receive remdesivir during the index hospitalization. Before the patient groups were balanced using IPTW, with respect to each characteristic individually, the majority of patients were 65 years or older, of non–Hispanic ethnicity, and approximately one-sixth of the overall population had an underlying immunocompromising condition. Also, before IPTW, patients treated with remdesivir vs those not treated with remdesivir were more likely to have received any supplemental oxygen during their index hospitalization (58.9% vs 42.4%), more likely to have been admitted to the ICU (20.2% vs 14.6%), and received LFO as their highest oxygen requirement (37.1% vs 28.6%) (Table 1). Before the 2 groups were balanced using IPTW, 86.8% of the patients who received remdesivir and 63.7% of patients who did not receive remdesivir received corticosteroid monotherapy during their index hospitalization. The patient population represented diverse types of hospital sizes with approximately half of the patient population in hospitals with bed size >300. Post-IPTW, approximately 69% of patients were elderly, and approximately 16% had an underlying immunocompromising condition (Table 1).

During the readmission, mean (standard deviation [SD]) duration of hospital stay was 7.6 (11.1) days, and 49% of patients were treated with corticosteroid monotherapy. During the Omicron period, the all-cause in-hospital mortality for the readmission was 21% (Table 2).

**Table 2.** Characteristics of Patients at COVID-19–Related Readmission After the Index hospitalization

		Overall^a^	Elderly^a^	Immunocompromised^a^
Primary diagnosis of COVID-19 at readmission, %		41	40	40
COVID-19 flagged as “Present on admission” at readmission, %		99	99	99
Primary or secondary diagnosis of pneumonia due to COVID-19 at readmission, %		53	52	55
Length of stay at readmission, d	Mean (SD)	7.6 (11.1)	7.6 (10.5)	8.9 (13.2)
	Median (Q1, Q3)	5.0 (3.0, 9.0)	6.0 (3.0, 10.0)	6.0 (3.0, 11.0)
COVID-19 treatment during the readmission, %	Remdesivir monotherapy	1	1	1
	Corticosteroids monotherapy	49	49	55
	Remdesivir + corticosteroids	9	9	12
	Remdesivir + corticosteroids/Baricitinib/tocilizumab	3	3	3
	Remdesivir + corticosteroids + Baricitinib/tocilizumab	5	5	5
All-cause in-hospital mortality, %		21	23	26

Abbreviations: COVID-19, coronavirus disease 2019; d, days; IPTW, inverse probability of treatment weighting; Q, quarter; SD, standard deviation.

^a^IPTW estimates are presented after trimming extreme propensity scores <0.05 and >0.95.

**Figure 2.**
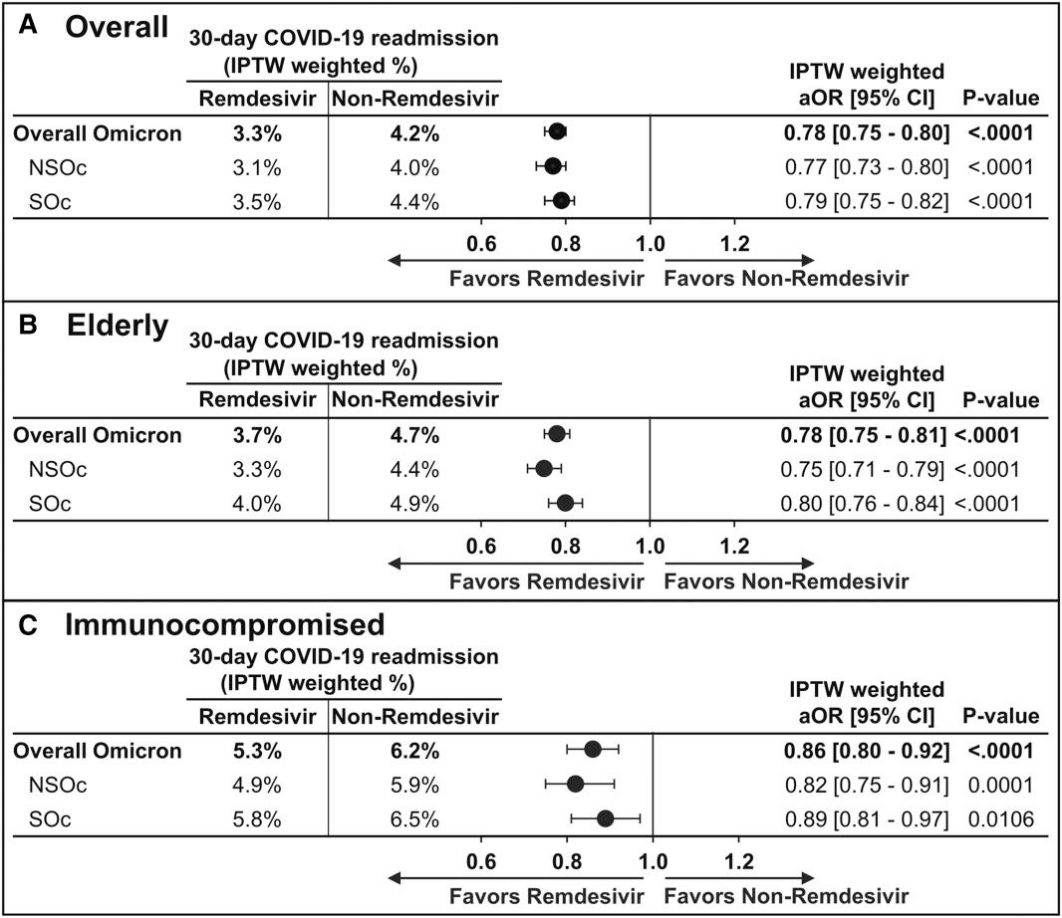
30-day COVID-19-related readmission among patients hospitalized for COVID-19 between December 2021 and February 2024 during Omicron era, by maximum supplemental oxygen requirement. (*A*), Overall population. (*B*), Elderly population. (*C*), Immunocompromised population. IPTW estimates after trimming extreme propensity scores <0.05 and >0.95. Abbreviations: aOR, adjusted odds ratio; CI, confidence interval; COVID-19, coronavirus disease 2019; IPTW, inverse probability of treatment weighting; NSOc, no supplemental oxygen charges; SOc, supplemental oxygen charges.

Lower odds of 30-day COVID-19-related readmission were observed in patients who received remdesivir vs those who did not receive remdesivir (3.3% vs 4.2%, respectively; OR [95% CI]: 0.78 [.75–.80], *P* < .0001), ie, 22% lower likelihood of 30-day COVID-19-related readmission in those treated with remdesivir during their index hospitalization for COVID-19 (Figure 2*A*). These results were consistent irrespective of supplemental oxygen requirement during the index hospitalization for COVID-19 who were discharged alive (OR [95% CI] for NSOc: 0.77 [.73–.80] and any SOc: 0.79 [.75–.82]) (Figure 2*A*).

### Elderly Population

In the elderly population discharged alive from the initial COVID-19 hospitalization, 74 892 (51.98%) patients were treated with remdesivir, and 69 200 (48.02%) patients did not receive remdesivir at index hospitalization. The mean (SD) age of patients who received remdesivir and those who did not receive remdesivir was 78.3 (7.5) and 78.4 (7.5) years, respectively. At index hospitalization, majority of the patients received corticosteroid monotherapy (85.1% of patients who received remdesivir and 62.6% of patients who did not receive remdesivir). Among patients requiring any supplemental oxygen, a higher proportion of patients treated with remdesivir vs those not treated with remdesivir at index hospitalization required HFO/NIV (17.1% vs 10.5%) and LFO (37.4% vs 29.6%), before the 2 groups were balanced using IPTW (Supplementary Table 2). Post–IPTW, the mean (SD) age of patients was 78.4 (10.4) years with approximately 27% of patients >85 years of age. More than 74% of patients received corticosteroid monotherapy, and approximately 16% of patients had an underlying immunocompromising condition at index hospitalization (Supplementary Table 2).

For the readmission, the mean (SD) length of stay was 7.6 (10.5) days with all-cause in-hospital mortality of 23%. Approximately half of patients (49%) were treated with corticosteroid monotherapy during the readmission (Table 2).

Patients who received remdesivir had a 22% lower likelihood of 30-day COVID-19-related readmission vs those who did not receive remdesivir (3.7% vs 4.7%, respectively; OR [95% CI]: 0.78 [.75–.81], *P* < .0001) (Figure 2*B*). These results were consistent irrespective of supplemental oxygen requirement (OR [95% CI] for NSOc: 0.75 [.71–.79] and any SOc: 0.80 [.76–.84]) (Figure 2*B*).

### Immunocompromised Population

In patients with an underlying immunocompromising condition, 18 542 (55.7%) patients were treated with remdesivir, and 14 775 (44.4%) patients did not receive remdesivir at index hospitalization. With respect to each individual assessment, most of these patients were 65 years or older (∼70%) and received corticosteroid monotherapy (71.1%–86.6%) at index hospitalization. Among those requiring any supplemental oxygen, a higher proportion of patients treated with remdesivir vs those not treated with remdesivir at index hospitalization required HFO/NIV (17.5% vs 11.9%) and LFO (35.4% vs 28.5%), respectively, before the 2 groups were balanced using IPTW (Supplementary Table 3). Post-IPTW, approximately 70% of the patients with an underlying immunocompromising condition were ≥65 years of age, and approximately 80% had received corticosteroid monotherapy at index hospitalization (Supplementary Table 3).

Upon readmission, patients with an underlying immunocompromising condition were in the hospital for a mean (SD) duration of 8.9 (13.2) days and the majority of patients were treated with corticosteroid monotherapy (55%). The all-cause in-hospital mortality of 26% for the readmission was reported in this vulnerable population (Table 2).

Patients who received remdesivir and were discharged alive from the initial COVID-19 hospitalization had a 14% lower likelihood of 30-day COVID-19-related readmission vs those who did not receive remdesivir (5.3% vs 6.2%, respectively; OR [95% CI]: 0.86 [.80–.92], *P* < .0001) (Figure 2*C*). Similar results were observed for NSOc and any supplemental oxygen groups (OR [95% CI] for NSOc: 0.82 [.75–.91] and any SOc: 0.89 [.81–.97]) (Figure 2*C*).

## DISCUSSION

Readmission rates in non-pandemic settings are increasingly used as quality-of-care indicators [11]. Considering the high logistical and financial burden on hospitals and patients associated with increasing rates of readmission, policymakers have identified 30-day hospital readmission as a key quality metric for assessing quality-of-care [40]. Findings from this real-world study of >200 000 patients hospitalized for COVID-19 indicated that many 30-day readmissions in patients hospitalized for COVID-19 (including vulnerable populations of older adults and those with an underlying immunocompromising condition) may be preventable, and that antiviral treatment with remdesivir could be an appropriate approach to improve overall outcomes including the key quality-of-care metric for readmission. Most importantly, as shown here, the clinical consequence of readmission within 30 days of discharge for a diagnosis for COVID-19 includes a 21% risk of in-hospital mortality (Table 2).

The comparative evidence derived from the IPTW approach in our observational study balance the treatment groups. In the current study, treatment with remdesivir was associated with a significantly lower likelihood of 30-day COVID-19-related hospital readmission and this finding was consistent across all subgroups and baseline supplemental oxygen requirements, including those without supplemental oxygen requirement, and across the spectrum of supplemental oxygen needs (HFO/NIV, LFO, and IMV/ECMO).

Early readmission after any cause of hospitalization is correlated with negative impact on patients’ quality of life and is associated with suboptimal outcomes and encumber a high economic burden. Hospital readmissions–related annual costs are more than 50 billion dollars [41], placing a significant economic burden on the healthcare system. With respect to hospitalization for COVID-19, a retrospective cohort study from a Boston-based, 673-bed academic medical center in patients admitted with COVID-19 between March to June 2020 showed that over 26.3% of 30-day hospital readmits were potentially preventable [42]. Understanding ways to prevent rehospitalization to eventually improve care services could enhance the quality of life of impacted individuals and minimize the burden on policymakers, healthcare system, and economy. Across a sample of recent studies, the 30-day readmission rate for COVID-19 hospitalizations ranged from 1.9% to 19.9% [7, 9, 10, 43–45]. In our study, the 30-day COVID-19-related readmissions in patients hospitalized for COVID-19 and treated with remdesivir was 3.3% and reflects the inclusion of more recent data in our sample that extends into the Omicron period, and improvement of patient outcomes over time. This finding, however, is similar albeit slightly improved in comparison to the previously reported 30-day COVID-19-related readmission rate of 3.6% [33].

Our findings build on evidence in selected smaller populations that characterize the impact of remdesivir on lowering risk of rehospitalization [8, 43, 46, 47]. A multicenter cohort study in Rhode Island, United States, including 2062 patients hospitalized for COVID-19 [46], showed that patients treated with remdesivir had a 19% decrease in the risk of 30-day readmission (95% CI: .59–1.13). Overall, the totality of the evidence highlights the benefit of treatment with remdesivir and a significant reduction in 30-day COVID-19-related hospital readmission. Nevertheless, one should exercise caution while comparing studies, considering the differences in study design, population cohort, outcomes, statistical considerations, geography, and study duration.

Findings from our study speak to the necessity of preventing hospital readmissions in patients with COVID-19. The mean LOS and high in-hospital mortality associated with hospital readmission, as observed in our study, reflect the complex nature of COVID-19, particularly in vulnerable populations. Patients readmitted after an initial hospitalization for COVID-19 have a potentially greater comorbidity burden and often experience severe health complications, leading to longer hospital stays and a high risk of mortality. Clinicians, hospitals, and policymakers should continually reevaluate the approach to minimize COVID-19-related readmissions within 30 days of index hospitalization. Using a geographically diverse sample across rural and urban hospitals, we could assess the apparent effectiveness of remdesivir in reducing hospital readmission, adding to the previous similar evidence [33]. Vulnerable patients with COVID-19, including the older adults (≥65 years of age) and those with underlying immunocompromising conditions are of particular interest as hospital readmissions in these groups encumber a substantial treatment and economic burden, and for many reasons, carries substantial risk of adverse health outcomes including mortality.

Older adults are at an elevated risk of initial or prolonged hospitalization due to COVID-19 and subsequent worse outcomes, including hospital readmissions and mortality [5]. Patients in our study were primarily elderly (∼68% of patients ≥65 years of age), in line with observations from a Premier Healthcare Database study in the United States from March to August 2020, highlighting the odds of readmission increasing in those aged ≥65 years [48]. In order to determine the effectiveness of remdesivir in reducing hospital readmission in older adults, we analyzed this sub-group separately. In the current analysis, only 3.7% of the older adults treated with remdesivir were readmitted to the hospital within 30 days of index hospitalization vs 4.7% of those not treated with remdesivir; translating to a 22% lower likelihood of 30-day COVID-19-related readmission in patients treated with remdesivir. Results across groups were consistent irrespective of oxygen requirement (including both non-hypoxemic and hypoxemic patients); patients treated with remdesivir and without need for supplemental oxygen had a 25% lower likelihood of 30-day COVID-19-related readmission, and those with any supplemental oxygen requirement had a 20% lower likelihood of readmission.

COVID-19 in patients with an underlying immunocompromising condition continues to incur risk, and the interaction between infection and the host immune system in such population remains poorly understood [49, 50]; paradoxically, such a population is often excluded or underrepresented in COVID-19 clinical trials [32, 51]. Remdesivir has demonstrated significant survival benefits across pre-Delta, Delta, and Omicron periods in immunocompromised patients hospitalized for COVID-19 [32]. In our study, among patients with COVID-19 and an underlying immunocompromising condition, treatment with remdesivir was associated with lower 30-day COVID-19-related readmission vs those not treated with remdesivir during their index hospitalization (5.3% vs 6.2%, respectively), similar to the previous evidence [32, 34, 36]. However, our findings are unique in assessing hospital readmission as they extend data to the current, Omicron, endemic era.

One of the key strengths of our study is incremental data highlighting the real-world benefit of remdesivir on COVID-19 hospital readmissions across the full spectrum of patient types, including the vulnerable populations hospitalized for COVID-19 across the United States in the recent Omicron-dominant era.

Similar to that described previously [33], our study was limited due to the possibility of residual confounding due to unmeasured differences between comparison groups. Our study investigated the impact of treatment choice on the risk of hospital readmission but was not designed to assess other contributing factors that warrant future research. Also, because the database does not capture readmission to a different hospital in the database or to a hospital not part of the database, it is likely that the readmission rates reported in this study are an underestimation of that observed in the real world. However, it is not expected that this would introduce any level of bias as there would not likely be a differential in readmissions to other hospitals between the treatment groups. It is likely that there would be a similar underestimation of readmissions to a different hospital in both the remdesivir and nonremdesivir treatment groups and will not impact the comparative likelihood of readmission for the 2 groups. Another limitation of this study is the lack of availability of important variables in the database such as vaccination status of patients and rate of previous infections. These unobserved factors may have affected physician's decisions to prescribe remdesivir leading to a differing proportion of vaccinated patients or patients with prior infection between the treatment groups. These unobserved differences between-group could potentially be responsible for the differential outcomes observed, which may be incorrectly attributed to remdesivir treatment leading to a biased estimate of benefit. In addition, vaccination status or prior infection could be effect modifiers for remdesivir further biasing the results if there are meaningful between-group differences in these factors. Although these data limitations do have the potential to introduce bias into our analysis, it is likely that any such bias would be minimal or would serve to underestimate the benefit of remdesivir. First, given that we are examining a group of high-risk patients, severe enough to be hospitalized for COVID-19, it reflects a potentially failed protection from prior immunity and hence, differences between patients with and without vaccination or prior infections and the associated biases would be minimal. In addition, it is expected that the PS matching approach which balanced the measured variables in this study (specifically age and key comorbidities) is likely to have potentially (at least partially) balanced out some of the unmeasured variables such as vaccination and prior infection as well. Finally, as clinicians may be more likely to prescribe remdesivir to those who are unvaccinated, such patients may be found in greater proportion in the remdesivir arm of the analysis. Given that we could expect worse outcomes among these patients owing to their unvaccinated status, this would serve to underestimate the benefit of remdesivir observed in the analysis. A further limitation is that post-discharge deaths occurring outside of the inpatient setting were not available in this database and hence, could not be accounted for during the follow-up period. This could have potentially led to between-group differences in the ability to observe the outcome of subsequent readmissions (ie, differential loss to follow-up). However, few post-discharge and outside-hospital deaths are expected within the follow-up period of 30 days. Lastly, our study focused on data collected during the Omicron–dominant period from December 2021 through February 2024, and findings may not be generalizable to other time periods or as other variants arise. Nonetheless, the findings are consistent with prior research.

## CONCLUSIONS

Treatment initiation with remdesivir during the index COVID-19 hospitalization was associated with significant benefit in reducing the 30-day readmissions across the overall population, older adults, and those with an underlying immunocompromising condition among patients discharged alive from the index COVID-19 hospitalization, irrespective of supplemental oxygen requirement. Our findings using a geographically diverse data set reinforce the continually growing body of evidence for the benefits of antiviral treatment with remdesivir. Here, we specifically describe the improved outcomes associated with remdesivir use with respect to the significantly reduced rehospitalization risk in vulnerable populations hospitalized for COVID-19. The evidence from this study adds reduced risk of rehospitalization as yet another rationale to consider remdesivir as the standard of care for the management of patients hospitalized for COVID-19.

## Supplementary Data

Supplementary materials are available at *Clinical Infectious Diseases* online. Consisting of data provided by the authors to benefit the reader, the posted materials are not copyedited and are the sole responsibility of the authors, so questions or comments should be addressed to the corresponding author.

## Supplementary Material

ciae511_Supplementary_Data

## Notes

***Author contributions.*** All authors contributed substantially to the manuscript and agreed to the final submitted version.

***Acknowledgments.*** All authors approved the final version of this article. The medical writing support was provided by Preetinder Kaur of Certara.

***Supplement sponsorship.*** This article appears as part of the supplement “Harnessing Real-world Data to Inform Clinical Practice: Lessons From the Evolving COVID-19 Era,” sponsored by Gilead Sciences, Inc.

***Data availability.*** The data supporting this study's findings are available from Premier, Inc (https://www.premierinc.com/). Restrictions apply to the availability of these data, which were used under license for this study.

***Ethics approval and consent to participate.*** This study did not require Ethics approval and informed consent. The analysis of data from the Premier Healthcare Database (US PINC AI Healthcare Database) was conducted under an exemption from the Institutional Review Board oversight for US-based studies using de-identified healthcare records that are not re-identifiable.

***Financial support.*** This study was supported by Gilead Sciences, Inc.

***Potential conflicts of interest.*** E. M., T. O., and M. B. report employment and being stockholders of Gilead Sciences during the conduct of the study. A. C. and H. J. report funding for the study and medical writing provided to their institution (Certara) from Gilead Sciences during the conduct of the study. R. L. G. has served on scientific advisory boards for AbbVie, AstraZeneca, Eli Lilly, Gilead Sciences, GSK, Roche, Johnson & Johnson (coronavirus disease 2019 [COVID-19]–related randomized clinical trial, coordinating principal investigator), and Kinevant Sciences (academic steering committee, study investigator; fees to Baylor Scott & White Research Institute); serving as a consultant for Gilead Sciences (honoraria for lectures), Johnson & Johnson, and Kinevant Sciences (through his institution); serving on a speaker bureau for Pfizer unrelated to COVID-19; his institution receiving a gift-in-kind from Gilead Sciences to facilitate a multicenter clinical trial outside the scope of COVID-19; a de minimis investment in AbCellera; grants or contracts as a study investigator (fees to Baylor Scott & White Research Institute) from Regeneron, Eli Lilly, Gilead Sciences, Pfizer, JNJ, AstraZeneca, and Roivant Sciences (Kinevant Sciences); and receipt of travel support for original scientific presentations from Gilead Sciences. A. C. K. reports grants from the National Institutes of Health Adaptive COVID-19 Treatment Trial. C. C. M. reports payment or honoraria for lectures/speakers from AstraZeneca, Boehringer Ingelheim, aTyr Pharma; consulting fees from AstraZeneca; and participation on advisory board for Gilead Sciences. A. N. A. has been a principal investigator or co-investigator of clinical trials sponsored by the National Institutes of Health/National Institute of Allergy and Infectious Diseases, NeuroRx Pharma, Pulmotect, Blade Therapeutics, Novartis, Takeda, Humanigen, Eli Lilly, PTC Therapeutics, OctaPharma, Fulcrum Therapeutics, and Alexion and a speaker and/or consultant for Pfizer, Salix, Alexion, AstraZeneca, Bayer, Ferring, Seres, Spero, Eli Lilly, Novo Nordisk, Gilead Sciences, Renibus, GSK, Dexcom, Reprieve, HeartRite, and Aseptiscope; these relationships were unrelated to the current work.
