# Supplementary material for: Remdesivir Effectiveness in Reducing the Risk of 30-Day Readmission in Vulnerable Patients Hospitalized for COVID-19: A Retrospective US Cohort Study Using Propensity Scores
Source: Clin Infect Dis. 2024 Oct 15;79(Suppl 4):S167–77. doi: 10.1093/cid/ciae511 (PMC11638780; doi:10.1093/cid/ciae511)
Supplement: ciae511_Supplementary_Data [file ciae511_supplementary_data.docx]

**Remdesivir Effectiveness in Reducing the Risk of 30-day Readmission in Vulnerable Patients Hospitalized for COVID-19: A Retrospective US Cohort Study Using Propensity Scores**

Essy Mozaffari^1^, Aastha Chandak^2^, Robert L. Gottlieb^3,4,5,6^, Andre C Kalil^7^, Heng Jiang^8^, Thomas Oppelt^1^, Mark Berry^9^, Chidinma Chima-Melton^10^, Alpesh N. Amin^11^

*^1^Medical Affairs, Gilead Sciences, Foster City, California, USA*

*^2^Evidence and Access, Certara, New York, New York, USA*

*^3^Department of Internal Medicine, Baylor University Medical Center, Dallas, Texas, USA*

*^4^Baylor Scott & White Heart and Vascular Hospital, Dallas, Texas, USA*

*^5^Baylor Scott & White The Heart Hospital, Plano, Texas, USA*

*^6^Baylor Scott & White Research Institute, Dallas, Texas, USA*

*^7^Division of Infectious Diseases, Department of Internal Medicine, University of Nebraska Medical Center, Omaha, Nebraska, USA*

*^8^Evidence and Access, Certara, Paris, France*

*^9^Real World Evidence, Gilead Sciences, Foster City, California, USA*

*^10^Pulmonary Division, Tele-ICU Inc, Los Angeles, California, USA*

*^11^Division of Hospital Medicine & Palliative Medicine, Department of Medicine, University of California Irvine, Orange, California, USA*

**Corresponding author:**

Alpesh N. Amin

Professor of Medicine, Business, Population & Public Health, Nursing Science, Pharmacy & Pharmaceutical Science, and Biomedical Engineering,

University of California Irvine, USA

Email: [anamin@uci.edu](mailto:anamin@uci.edu)

# SUPPLEMENTARY TABLES AND FIGURES

## Supplementary Table 1: Study covariates included in the logistic regression model

| Key Study Covariates | | Definitions |
| --- | --- | --- |
| Supplemental Oxygen  Requirements | IMV/ECMO | Billing charges: Invasive mechanical ventilation, tracheostomy, endotracheal tube, intubation, extracorporeal membrane oxygenation |
|  | HFO/NIV | Billing charges: Negative-pressure ventilation, positive-pressure ventilation, CPAP, BiPAP, high flow system via nasal cannula, venturi face mask, rebreather, non-rebreather mask, positive expiratory pressure |
|  | LFO | Billing charges: Simple face mask, oxygen pendant, low flow system via nasal cannula, oxygen supply |
|  | NSOc | No billing charges for IMV/ECMO, HFO/NIV, or LFO at baseline (defined above) |
| Use of corticosteroids |  | Billing charges for treatment: Prednisone, prednisolone, methylprednisolone, hydrocortisone, dexamethasone, |
| Comorbid conditions of interest | Cancer | ICD-10-CM diagnosis codes: C00-C96 |
|  | Immunocompromising conditions | ICD-10-CM code for cancer (C00-C96), transplant (Z94.x), hematologic malignancies (C81.x, C82.x, C83.x, C84.x, C85.x, C88.x, C90.x, C91.x, C92.x, C93.x, C94.x, C95.x, C96.x), primary immunodeficiencies (D80.x, D81.x, D82.x, D83.x, D84.x, G11.3, E70.330, D71.x, D70.x), asplenia (Q89.01, Z90.81), toxic effects of antineoplastics (T45.1x), bone marrow failure/aplastic anemia (D61.x), severe combined immunodeficiencies (D80.x, D81.x, D82.x, D83.x, D84.x, D86.x, D89.0, D89.1, D89.2, D89.3, D89.4x, D89.81, D89.82, D89.89, D89.9), HIV (B20), patients with chronic graft-versus-host disease or who are taking immunosuppressive medications for another indication (Z89.8x, Z79.52, Z79.61, Z79.62x, Z79.63x, Z79.64, Z79.69, Z79.810, Z79.811, Z79.818) |
|  | Obesity | ICD-10-CM diagnosis codes: E66, Z6825-Z6845 |
|  | Chronic obstructive pulmonary disorder | ICD-10-CM diagnosis codes: I278, I279, J40, J41, J42, J43, J44, J45, J46, J47, J60, J61, J62, J63, J64, J65, J66, J67, J684, J701, J703 |
|  | Cardiovascular disease (including hypertension) | ICD-10-CM diagnosis codes: I00-I99 |
|  | Diabetes mellitus | ICD-10-CM diagnosis codes: E10-E14 |
|  | Renal disease | ICD-10-CM diagnosis codes: I120, I131, N032, N033, N034, N035, N036, N037, N052, N053, N054, N055, N056, N057, N18, N19, N250, Z490, Z491, Z492, Z940, Z992 |

Abbreviations: HFO*/*NIV, high-flow oxygen*/*non-invasive ventilation; HIV, human immunodeficiency virus ICD-10-CM, International Classification of Diseases, 10^th^ revision, Clinical Modification; IMV*/*ECMO, invasive mechanical ventilation*/*extracorporeal membrane oxygenation; LFO, low-flow oxygen; NSOc, no supplementary oxygen charges.

## Supplementary Table 2: Characteristics of patients during the index COVID-19 hospitalization in the elderly population (before and after IPTW)

|  | **Before IPTW** | | | **After IPTW*** | | |
| --- | --- | --- | --- | --- | --- | --- |
|  | **Non-Remdesivir** | **Remdesivir** | **SMD** | **Non-Remdesivir** | **Remdesivir** | **SMD** |
| **Number of patients** | **n=69,200** | **n=74,892** |  | **-** | **-** | **-** |
| **Number of hospitals** | **n=901** | **n=898** |  | **-** | **-** | **-** |
| **Age, years (not included in PS calculation)** |  |  |  |  |  |  |
| Mean (SD) | 78.4 (7.5) | 78.3 (7.5) | −0.02 | 78.3 (10.9) | 78.4 (10.4) | 0.01 |
| Median (Q1, Q3) | 79.0 (72.0, 85.0) | 78.0 (72.0, 85.0) |  | 78 (72.0, 85.0) | 78 (72.0, 85.0) |  |
| **Age group, years** |  |  |  |  |  |  |
| 65–74 | 23,531 (34.0) | 26,178 (35.0) | 0.02 | 34.6 | 34.5 | 0.03 |
| 75–84 | 26,863 (38.8) | 28,812 (38.5) |  | 38.6 | 38.6 |  |
| ≥85 | 18,806 (27.2) | 19,902 (26.6) |  | 26.8 | 26.9 |  |
| **Race** |  |  |  |  |  |  |
| White | 54,772 (79.2) | 60,427 (80.7) | 0.10 | 79.9 | 79.9 | 0.00 |
| Black | 8,553 (12.4) | 7,637 (10.2) |  | 11.3 | 11.3 |  |
| Asian | 1,388 (2.0) | 1,992 (2.7) |  | 2.4 | 2.3 |  |
| Other | 4,487 (6.5) | 4,836 (6.5) |  | 6.5 | 6.4 |  |
| **Gender** |  |  |  |  |  |  |
| Female | 36,538 (52.8) | 38,861 (51.9) | 0.04 | 52.1 | 52.1 | 0.00 |
| **Ethnicity** |  |  |  |  |  |  |
| Hispanic | 4,619 (6.7) | 6,827 (9.1) | 0.11 | 7.7 | 7.8 | 0.00 |
| Non-Hispanic | 59,464 (85.9) | 64,044 (85.5) |  | 86.0 | 86.0 |  |
| Unknown | 5,117 (7.4) | 4,021 (5.4) |  | 6.2 | 6.2 |  |
| **CCI** |  |  |  |  |  |  |
| 0 | 9,359 (13.5) | 9,481 (12.7) | 0.06 | 12.9 | 12.9 | 0.00 |
| 1–3 | 37,304 (53.9) | 42,400 (56.6) |  | 55.2 | 55.1 |  |
| ≥4 | 22,537 (32.6) | 23,011 (30.7) |  | 31.9 | 32.0 |  |
| **Comorbid conditions** |  |  |  |  |  |  |
| Immunocompromising condition | 10,325 (14.9) | 13,021 (17.4) | 0.07 | 16.4 | 16.5 | 0.00 |
| Cancer | 4,613 (6.7) | 5,972 (8.0) | 0.05 | 7.4 | 7.4 | 0.00 |
| Obesity | 14143 (20.4) | 16544 (22.1) | 0.04 | 21.4 | 21.4 | 0.00 |
| COPD | 22781 (32.9) | 29203 (39.0) | 0.13 | 36.3 | 36.3 | 0.00 |
| Cardiovascular disease | 64012 (92.5) | 69053 (92.2) | −0.01 | 92.4 | 92.4 | 0.00 |
| Diabetes mellitus | 27190 (39.3) | 28951 (38.7) | −0.01 | 39.1 | 39.0 | 0.00 |
| Renal disease | 23302 (33.7) | 21347 (28.5) | −0.11 | 31.3 | 31.4 | 0.00 |
| **Maximum supplemental oxygen requirement** |  |  |  |  |  |  |
| IMV/ECMO | 1,111 (1.6) | 1,420 (1.9) | 0.27 | 1.8 | 1.8 | 0.00 |
| HFO/NIV | 7,281 (10.5) | 12,833 (17.1) |  | 14.2 | 14.1 |  |
| LFO | 20,496 (29.6) | 28,002 (37.4) |  | 33.6 | 33.8 |  |
| NSOc | 40,312 (58.3) | 32,637 (43.6) |  | 50.5 | 50.4 |  |
| **ICU use** | 9,282 (13.4) | 14,635 (19.5) | 0.16 | 16.7 | 16.8 | 0.00 |
| **Use of corticosteroids** | 43,329 (62.6) | 63,719 (85.1) | −0.03 | 74.4 | 74.7 | 0.00 |
| **Hospital setting** |  |  |  |  |  |  |
| Rural | 9,686 (14.0) | 8,943 (11.9) | 0.05 | 13.0 | 12.8 | 0.00 |
| Urban | 59,514 (86.0) | 65,949 (88.1) |  | 87.0 | 87.2 |  |
| **Hospital teaching status** | 26,929 (38.9) | 31,829 (42.5) | 0.06 | 40.8 | 40.9 | 0.00 |
| **Hospital census region** |  |  |  |  |  |  |
| Midwest | 17,562 (25.4) | 17,920 (23.9) | 0.15 | 25.0 | 25.0 | 0.00 |
| Northeast | 8,458 (12.2) | 12,689 (16.9) |  | 14.7 | 14.7 |  |
| South | 36,041 (52.1) | 34,992 (46.7) |  | 48.8 | 48.7 |  |
| West | 7,139 (10.3) | 9,291 (12.4) |  | 11.6 | 11.6 |  |
| **Hospital bed size** |  |  |  |  |  |  |
| <100 | 6,485 (9.4) | 6,308 (8.4) | 0.11 | 9.0 | 8.9 | 0.00 |
| 100–199 | 12,043 (17.4) | 13,320 (17.8) |  | 17.4 | 17.4 |  |
| 200–299 | 15,046 (21.7) | 14,972 (20.0) |  | 20.7 | 20.9 |  |
| 300–399 | 12,691 (18.3) | 11,904 (15.9) |  | 16.8 | 16.8 |  |
| 400–499 | 7,590 (11.0) | 8,194 (10.9) |  | 11.2 | 11.3 |  |
| ≥500 | 15,345 (22.2) | 20,194 (27.0) |  | 24.8 | 24.8 |  |
| **Admission month** |  |  | 0.14 |  |  | 0.00 |
| Dec 2021 | 5295 (7.7) | 6783 (9.1) |  | 8.3 | 8.2 |  |
| Jan 2022 | 12019 (17.4) | 12514 (16.7) |  | 16.8 | 16.6 |  |
| Feb 2022 | 3597 (5.2) | 3180 (4.2) |  | 4.7 | 4.7 |  |
| Mar 2022 | 724 (1.0) | 628 (0.8) |  | 0.9 | 0.9 |  |
| Apr 2022 | 772 (1.1) | 858 (1.1) |  | 1.1 | 1.1 |  |
| May 2022 | 2011 (2.9) | 2305 (3.1) |  | 2.9 | 2.9 |  |
| Jun 2022 | 2758 (4.0) | 2845 (3.8) |  | 3.8 | 3.8 |  |
| Jul 2022 | 3947 (5.7) | 3918 (5.2) |  | 5.4 | 5.4 |  |
| Aug 2022 | 3426 (5.0) | 3310 (4.4) |  | 4.7 | 4.7 |  |
| Sep 2022 | 2421 (3.5) | 2328 (3.1) |  | 3.3 | 3.4 |  |
| Oct 2022 | 2092 (3.0) | 2180 (2.9) |  | 3.0 | 3.0 |  |
| Nov 2022 | 2353 (3.4) | 2479 (3.3) |  | 3.4 | 3.4 |  |
| Dec 2022 | 3816 (5.5) | 4232 (5.7) |  | 5.6 | 5.6 |  |
| Jan 2023 | 3145 (4.5) | 3224 (4.3) |  | 4.4 | 4.5 |  |
| Feb 2023 | 1897 (2.7) | 2101 (2.8) |  | 2.8 | 2.8 |  |
| Mar 2023 | 1599 (2.3) | 1740 (2.3) |  | 2.3 | 2.4 |  |
| Apr 2023 | 1101 (1.6) | 1183 (1.6) |  | 1.6 | 1.6 |  |
| May 2023 | 794 (1.1) | 941 (1.3) |  | 1.2 | 1.2 |  |
| Jun 2023 | 635 (0.9) | 672 (0.9) |  | 0.9 | 0.9 |  |
| Jul 2023 | 916 (1.3) | 1000 (1.3) |  | 1.3 | 1.3 |  |
| Aug 2023 | 1862 (2.7) | 2113 (2.8) |  | 2.8 | 2.8 |  |
| Sep 2023 | 2107 (3.0) | 2343 (3.1) |  | 3.2 | 3.2 |  |
| Oct 2023 | 1659 (2.4) | 1886 (2.5) |  | 2.5 | 2.5 |  |
| Nov 2023 | 1832 (2.6) | 2242 (3.0) |  | 2.9 | 2.9 |  |
| Dec 2023 | 2923 (4.2) | 3654 (4.9) |  | 4.6 | 4.7 |  |
| Jan 2024 | 2370 (3.4) | 2885 (3.9) |  | 3.7 | 3.7 |  |
| Feb 2024 | 1129 (1.6) | 1348 (1.8) |  | 1.7 | 1.8 |  |
| **Length of stay, days (not included in PS calculation)** |  |  |  |  |  |  |
| Mean (SD) | 6.0 (12.7) | 7.1 (11.3) | 0.09 | 6.3 (20.9) | 6.8 (14.9) | 0.03 |
| Median (Q1, Q3) | 4.0 (2.0, 7.0) | 5.0 (3.0, 8.0) |  | 4.0 (2.0, 7.0) | 5.0 (3.0, 8.0) |  |
| **Remdesivir treatment duration, days (not included in PS calculation)** |  |  |  |  |  |  |
| Mean (SD) | - | 5.4 (2.3) | - | - | 5.3 (3.2) | - |
| Median (Q1, Q3) | - | 5 (4.0, 6.0) | - | - | 5 (4.0, 6.0) | - |
| **Hospital day of remdesivir initiation, days (not included in PS calculation)** |  |  |  |  |  |  |
| Mean (SD) | - | 1.4 (0.9) | - | - | 1.4 (1.2) | - |
| Median (Q1, Q3) | - | 1.0 (1.0, 2.0) | - | - | 1.0 (1.0, 2.0) | - |
| * After trimming extreme propensity scores <0.05 and >0.95  Data are presented as n (%) before IPTW and as % after IPTW, unless otherwise indicated.  Abbreviations: CCI, Charlson Comorbidity Index; COPD, chronic obstructive pulmonary disorder; COVID-19, coronavirus disease; ECMO, extracorporeal membrane oxygenation; HFO, High-flow oxygen; ICU, intensive care unit; IMV, invasive mechanical ventilation; IPTW, inverse probability treatment weighting; LFO, low-flow oxygen; NIV, non-invasive ventilation; NSOc, no supplementary oxygen charges; PS, propensity score; SD, standard deviation; SMD, standardized mean difference. | | | | | | |

## Supplementary Table 3: Characteristics of patients during the index COVID-19 hospitalization in immunocompromised population (before and after IPTW)

|  | **Before IPTW** | | | **After IPTW*** | | |
| --- | --- | --- | --- | --- | --- | --- |
|  | **Non-Remdesivir** | **Remdesivir** | **SMD** | **Non-Remdesivir** | **Remdesivir** | **SMD** |
| **Number of patients** | **n=14,775** | **n=18,542** |  | **-** | **-** | **-** |
| **Number of hospitals** | **n=792** | **n=803** |  | **-** | **-** | **-** |
| **Age, years (not included in PS calculation)** |  |  |  |  |  |  |
| Mean (SD) | 70.0 (14.0) | 70.1 (13.8) | 0.01 | 70.0 (20.7) | 70 (18.6) | 0.00 |
| Median (Q1, Q3) | 72.0 (62.0, 80.0) | 72.0 (62.0, 80.0) |  | 72.0 (62.0, 80.0) | 72.0 (62.0, 80.0) |  |
| **Age group, years** |  |  |  |  |  |  |
| 18-49 | 1,286 (8.7) | 1,501 (8.1) | 0.04 | 8.4 | 8.4 | 0.00 |
| 50-64 | 3,164 (21.4) | 4,020 (21.7) |  | 21.6 | 21.5 |  |
| ≥65 | 10,325 (69.9) | 13,021 (70.2) |  | 70.1 | 70.1 |  |
| **Race** |  |  |  |  |  |  |
| White | 11,155 (75.5) | 14,081 (75.9) | 0.06 | 75.8 | 75.7 | 0.00 |
| Black | 2,401 (16.3) | 2,630 (14.2) |  | 15.1 | 15.2 |  |
| Asian | 237 (1.6) | 438 (2.4) |  | 2.0 | 2.0 |  |
| Other | 982 (6.6) | 1,393 (7.5) |  | 7.1 | 7.1 |  |
| **Gender** |  |  |  |  |  |  |
| Female | 7,479 (50.6) | 9,546 (51.5) | 0.01 | 51.1 | 51.0 | 0.00 |
| **Ethnicity** |  |  |  |  |  |  |
| Hispanic | 1,189 (8.0) | 2,105 (11.4) | 0.12 | 9.6 | 9.8 | 0.00 |
| Non-Hispanic | 12,471 (84.4) | 15,472 (83.4) |  | 84.2 | 84.1 |  |
| Unknown | 1,115 (7.5) | 965 (5.2) |  | 6.1 | 6.1 |  |
| **CCI** |  |  |  |  |  |  |
| 0 | 1,131 (7.7) | 1,246 (6.7) | 0.06 | 7.1 | 7.2 | 0.00 |
| 1–3 | 6,564 (44.4) | 8,733 (47.1) |  | 45.9 | 45.9 |  |
| ≥4 | 7,080 (47.9) | 8,563 (46.2) |  | 47.0 | 46.9 |  |
| **Comorbid conditions** |  |  |  |  |  |  |
| Immunocompromising condition | 14,775 (100.0) | 18,542 (100.0) | 0.00 | 100.0 | 100.0 | 0.00 |
| Cancer | 5,906 (40.0) | 7,684 (41.4) | 0.03 | 40.7 | 40.6 | 0.00 |
| Obesity | 3421 (23.2) | 4567 (24.6) | 0.03 | 24.1 | 24 | 0.00 |
| COPD | 5259 (35.6) | 7555 (40.7) | 0.11 | 38.6 | 38.5 | 0.00 |
| Cardiovascular disease | 12834 (86.9) | 16107 (86.9) | 0.00 | 86.8 | 86.8 | 0.00 |
| Diabetes mellitus | 5385 (36.4) | 6659 (35.9) | −0.01 | 36.2 | 36.1 | 0.00 |
| Renal disease | 5215 (35.3) | 5635 (30.4) | −0.10 | 32.7 | 32.7 | 0.00 |
| **Maximum supplemental oxygen requirement** |  |  |  |  |  |  |
| IMV/ECMO | 330 (2.2) | 458 (2.5) | 0.25 | 2.4 | 2.4 | 0.00 |
| HFO/NIV | 1,759 (11.9) | 3,247 (17.5) |  | 15.3 | 15.1 |  |
| LFO | 4,213 (28.5) | 6,570 (35.4) |  | 32.2 | 32.3 |  |
| NSOc | 8,473 (57.3) | 8,267 (44.6) |  | 50.2 | 50.2 |  |
| **ICU use** | 2,187 (14.8) | 3,892 (21.0) | 0.16 | 18.2 | 18.3 | 0.00 |
| **Use of corticosteroids** | 10,499 (71.1) | 16,062 (86.6) | −0.02 | 79.8 | 79.9 | 0.00 |
| **Hospital setting** |  |  |  |  |  |  |
| Rural | 1,823 (12.3) | 1,895 (10.2) | 0.03 | 11.1 | 11.0 | 0.00 |
| Urban | 12,952 (87.7) | 16,647 (89.8) |  | 88.9 | 89.0 |  |
| **Hospital teaching status** | 6,112 (41.4) | 8,617 (46.5) | 0.03 | 44.2 | 44.3 | 0.00 |
| **Hospital census region** |  |  |  |  |  |  |
| Midwest | 3,674 (24.9) | 4,340 (23.4) | 0.16 | 24.3 | 24.1 | 0.03 |
| Northeast | 1,681 (11.4) | 2,948 (15.9) |  | 13.7 | 13.8 |  |
| South | 7,987 (54.1) | 9,217 (49.7) |  | 51.5 | 51.5 |  |
| West | 1,433 (9.7) | 2,037 (11.0) |  | 10.5 | 10.5 |  |
| **Hospital bed size** |  |  |  |  |  |  |
| <100 | 1,158 (7.8) | 1,355 (7.3) | 0.16 | 7.7 | 7.6 | 0.00 |
| 100–199 | 2,450 (16.6) | 3,016 (16.3) |  | 16.0 | 16.1 |  |
| 200–299 | 2,999 (20.3) | 3,341 (18.0) |  | 19.0 | 19.0 |  |
| 300–399 | 2,697 (18.3) | 2,881 (15.5) |  | 16.6 | 16.6 |  |
| 400–499 | 1,737 (11.8) | 2,066 (11.1) |  | 11.8 | 11.7 |  |
| ≥500 | 3,734 (25.3) | 5,883 (31.7) |  | 28.9 | 28.9 |  |
| **Admission month** |  |  | 0.33 |  |  | 0.00 |
| Dec 2021 | 1249 (8.5) | 1724 (9.3) |  | 8.9 | 8.8 |  |
| Jan 2022 | 2735 (18.5) | 3226 (17.4) |  | 17.9 | 17.7 |  |
| Feb 2022 | 841 (5.7) | 903 (4.9) |  | 5.2 | 5.2 |  |
| Mar 2022 | 222 (1.5) | 213 (1.1) |  | 1.3 | 1.3 |  |
| Apr 2022 | 166 (1.1) | 201 (1.1) |  | 1.1 | 1.1 |  |
| May 2022 | 425 (2.9) | 590 (3.2) |  | 2.9 | 3.0 |  |
| Jun 2022 | 595 (4.0) | 679 (3.7) |  | 3.8 | 3.8 |  |
| Jul 2022 | 829 (5.6) | 917 (4.9) |  | 5.2 | 5.2 |  |
| Aug 2022 | 740 (5.0) | 747 (4.0) |  | 4.5 | 4.5 |  |
| Sep 2022 | 513 (3.5) | 579 (3.1) |  | 3.3 | 3.3 |  |
| Oct 2022 | 401 (2.7) | 537 (2.9) |  | 2.8 | 2.9 |  |
| Nov 2022 | 468 (3.2) | 592 (3.2) |  | 3.2 | 3.3 |  |
| Dec 2022 | 760 (5.1) | 979 (5.3) |  | 5.1 | 5.2 |  |
| Jan 2023 | 608 (4.1) | 810 (4.4) |  | 4.2 | 4.3 |  |
| Feb 2023 | 394 (2.7) | 537 (2.9) |  | 2.8 | 2.8 |  |
| Mar 2023 | 342 (2.3) | 396 (2.1) |  | 2.2 | 2.2 |  |
| Apr 2023 | 231 (1.6) | 276 (1.5) |  | 1.5 | 1.5 |  |
| May 2023 | 181 (1.2) | 256 (1.4) |  | 1.3 | 1.3 |  |
| Jun 2023 | 141 (1.0) | 172 (0.9) |  | 0.9 | 1.0 |  |
| Jul 2023 | 214 (1.4) | 243 (1.3) |  | 1.4 | 1.4 |  |
| Aug 2023 | 380 (2.6) | 541 (2.9) |  | 2.7 | 2.8 |  |
| Sep 2023 | 372 (2.5) | 558 (3.0) |  | 2.8 | 2.8 |  |
| Oct 2023 | 330 (2.2) | 440 (2.4) |  | 2.4 | 2.4 |  |
| Nov 2023 | 371 (2.5) | 507 (2.7) |  | 2.7 | 2.7 |  |
| Dec 2023 | 561 (3.8) | 843 (4.5) |  | 4.2 | 4.3 |  |
| Jan 2024 | 489 (3.3) | 749 (4.0) |  | 3.8 | 3.8 |  |
| Feb 2024 | 217 (1.5) | 327 (1.8) |  | 1.6 | 1.6 |  |
| **Length of stay, days (not included in PS calculation)** |  |  |  |  |  |  |
| Mean (SD) | 6.4 (15.3) | 7.4 (11.5) | 0.08 | 6.8 (25.3) | 7.2 (14.8) | 0.02 |
| Median (Q1, Q3) | 4.0 (2.0, 7.0) | 5.0 (3.0, 8.0) |  | 4.0 (2.0, 7.0) | 5.0 (3.0, 8.0) |  |
| **Remdesivir treatment duration, days (not included in PS calculation)** |  |  |  |  |  |  |
| Mean (SD) | - | 5.6 (2.4) | - | - | 5.5 (3.2) | - |
| Median (Q1, Q3) | - | 6.0 (4.0, 6.0) | - | - | 5.0 (4.0, 6.0) | - |
| **Hospital day of remdesivir initiation, days (not included in PS calculation)** |  |  |  |  |  |  |
| Mean (SD) |  | 1.5 (1.0) | - | - | 1.5 (1.3) |  |
| Median (Q1, Q3) |  | 1.0 (1.0, 2.0) | - | - | 1.0 (1.0, 2.0) |  |
| * After trimming extreme propensity scores <0.05 and >0.95.  Data are presented as n (%) before IPTW and as % after IPTW, unless otherwise indicated.  Abbreviations: CCI, Charlson Comorbidity Index; COPD, chronic obstructive pulmonary disorder; COVID-19, coronavirus disease; ECMO, extracorporeal membrane oxygenation; HFO, High-flow oxygen; ICU, intensive care unit; IMV, Invasive mechanical ventilation; IPTW, inverse probability treatment weighting; LFO, low-flow oxygen; NIV, non-invasive ventilation; NSOc, no supplementary oxygen charges; PS, propensity score SD, standard deviation; SMD, standardized mean difference. | | | | | | |

## Supplementary Figure 1. Balance of standardized mean difference before and after IPTW in overall population


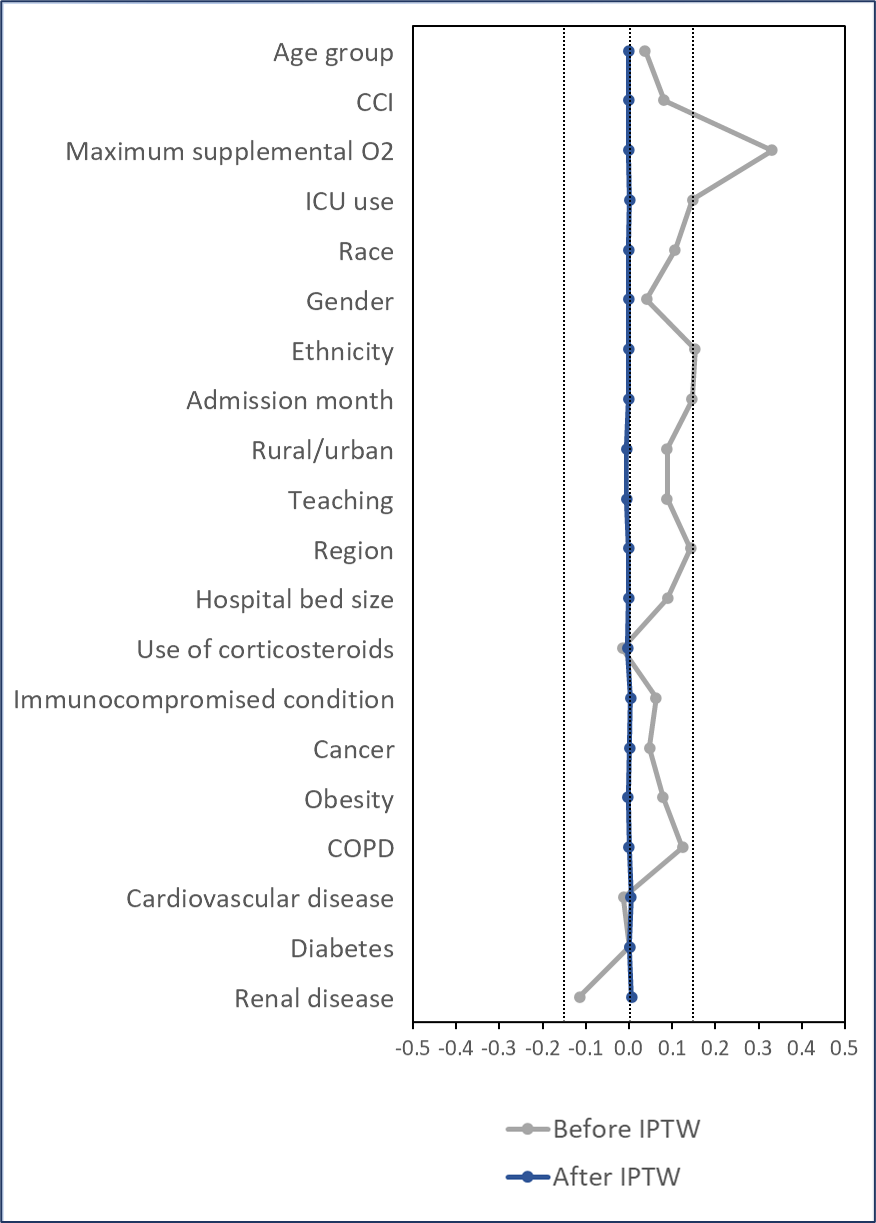


Abbreviations: CCI, Charlson Comorbidity Index; COPD, chronic obstructive pulmonary disorder; ICU, intensive care unit; IPTW, inverse probability of treatment weighting; O2, oxygen.

## Supplementary Figure 2. Balance of standardized mean difference before and after IPTW in the elderly population


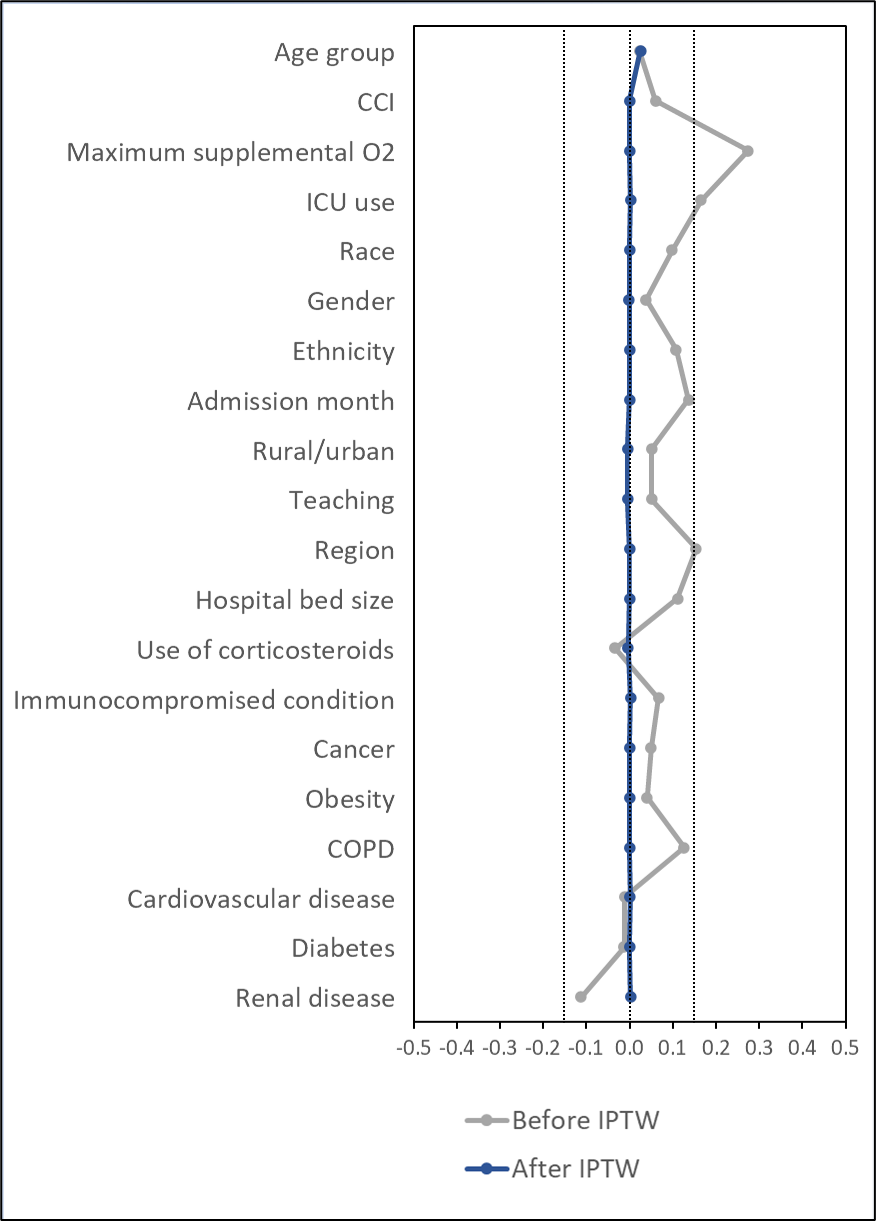


Abbreviations: CCI, Charlson Comorbidity Index; COPD, chronic obstructive pulmonary disorder; ICU, intensive care unit; IPTW, inverse probability of treatment weighting; O2, oxygen.

## Supplementary Figure 3. Balance of standardized mean difference before and after IPTW in the immunocompromised population


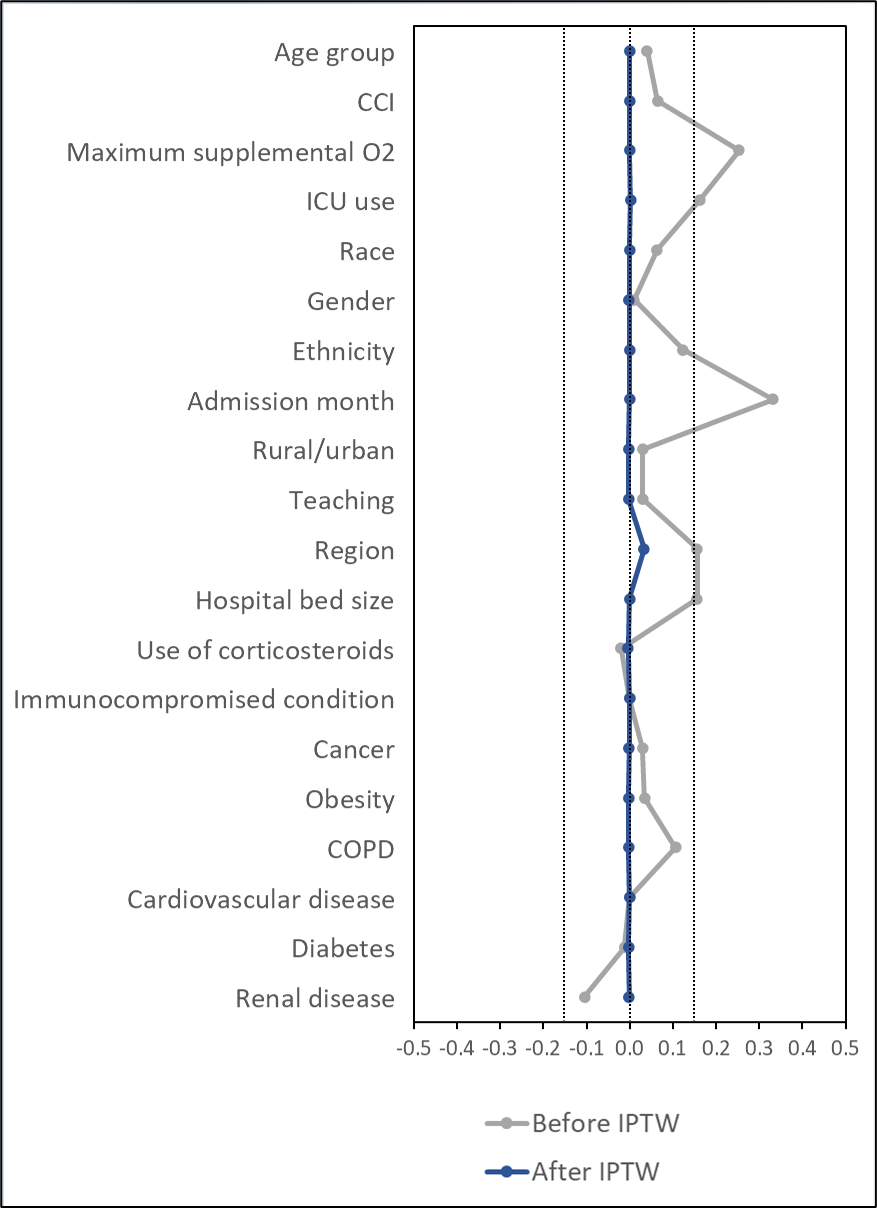


Abbreviations: CCI, Charlson Comorbidity Index; COPD, chronic obstructive pulmonary disorder; ICU, intensive care unit; IPTW, inverse probability of treatment weighting; O2, oxygen.
